# Adenoid Cystic Carcinoma of Salivary Glands: A Ten-Year Review and an Assessment of the Current Management, Surgery, Radiotherapy, and Chemotherapy

**DOI:** 10.1155/2023/7401458

**Published:** 2023-04-29

**Authors:** Eyad Saleh, Abdouldaim Ukwas

**Affiliations:** Eastman Dental Institute, University College London, London, UK

## Abstract

Adenoid cystic carcinoma (ACC) is a rare cancer that arises from the salivary glands and other sites in the body, such as the lung and breast. Although the tumor accounts for 10% of all salivary gland malignancies, it only accounts for 1% of head and neck malignancies. It can affect both major and minor salivary glands; here, it is called salivary gland adenoid cystic carcinoma or SACC, with a slight predilection to the latter, and commonly manifests between the 6_th_ and 7_th_ decades of life. The disease also shows a slight female predilection, with a reported female to male ratio of 3 : 2. Lesions of SACC are often insidious and slow-growing, and symptoms such as pain and altered sensation are frequently associated with advanced stages of the disease. Salivary adenoid cystic carcinoma is characterized by perineural invasion (PNI), a distinctive feature that potentially plays a significant role in the tumor's relapse and recurrence, which is approximately 50%. The disease is not prevalent, and its etiopathogenesis is poorly understood, although several genetic patterns and biomarkers have been linked to its initiation and/or progression. The discovery of these mutations and biomarkers has encouraged several clinical studies to use therapeutic agents to target the specific receptors on the cancer cells to potentially prevent further proliferation of the tumor cells and metastasis of the disease. Diagnosis of SACC is often challenging and frequently requires a combination of clinical examination, imaging, and histopathology. Management of SACC is primarily surgical excision, while radiotherapy has shown to be effective in improving local control in cases with microscopic residual disease. However, treatment of recurrent or metastatic tumors by radiotherapy with or without chemotherapy has so far shown limited success. The aim of this thesis is to provide an update of literature on SACC with a particular focus on the latest management approaches and future trends.

## 1. Introduction

Adenoid cystic carcinoma (ACC) is a rare malignancy that originates from the salivary glands and other sites in the body, such as the lung and breast. The tumor affects both major and minor salivary glands, with a slight predilection to the latter. It accounts for 10% of all salivary gland neoplasms and approximately 1% of head and neck malignancies. Although SACC can affect all age groups, it commonly manifests between the sixth and seventh decades of life. Moreover, SACC shows a slight predilection for women, with a reported female-to-male ratio of 3 : 2. Lesions are often slow-growing and asymptomatic, especially in the early stages, while pain and altered sensation are frequently associated with advanced stages of the disease. Adenoid cystic carcinoma is characterized by per neural invasion (PNI), a distinctive feature that facilitates its local and systematic spread and potentially plays a significant role in the tumor's relapse and recurrence, which is approximately 50%. The accurate etiology of SACC is poorly understood, but several studies have identified a number of genetic mutations which could potentially be involved in its carcinogenesis. The discovery of these mutations has encouraged several clinical studies to use therapeutic agents such as multitargeted tyrosine kinase inhibitors (TKIs) to target the same receptors on the cancer cells to potentially prevent further proliferation of the tumor cells and metastasis of the disease. Management of SACC is primarily surgical, with wide excision still considering the treatment of choice, while neck dissection is often indicated with positive lymph nodes. Treatment of recurrent or metastatic tumors by radiotherapy with or without chemotherapy has so far shown limited success. Nevertheless, radiotherapy has shown to be effective in improving local control in cases with microscopic residual disease.

## 2. Methodology

**Figure 1:**
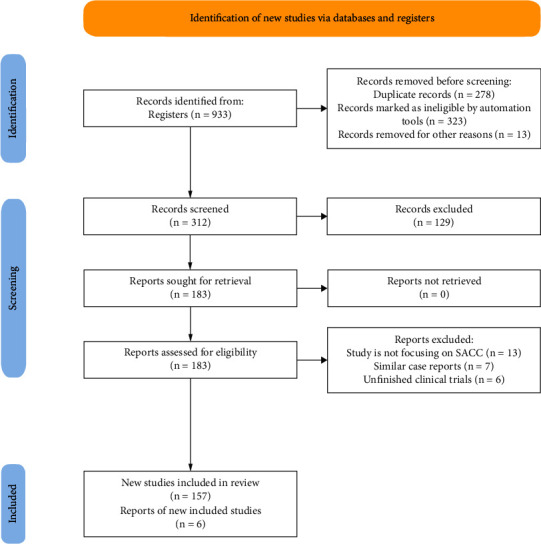
SACC search strategy.

Two databases were searched: PubMed/National Library of Medicine (NLM) and Cochrane Library. These databases were searched from August 01_st_, 2011 to August 31_st_, 2021, using the search terms “adenoid cystic carcinoma and salivary glands,” “salivary adenoid cystic carcinoma,” and “adenoid cystic carcinoma of salivary glands.” Exclusion criteria included titles which include one term without the other, and ACC of sites out of the scope of this review, i.e., only salivary glands lesions were included. The following flowchart shows the search strategy Figure 1.

## 3. Results

PubMed results included literature reviews, systemic reviews, case reports, case series, and retrospective studies, and they were as follows: 640 articles for the search terms “adenoid cystic carcinoma and salivary glands,” 191 articles for “salivary adenoid cystic carcinoma,” and 82 articles for “adenoid cystic carcinoma of salivary glands.” Results from the Cochrane Library search were 22 articles, of which 9 were clinical trials. A total number of 278 duplicate records and 323 ineligible reports were removed. The initial records of 312 were assessed primarily, and further 129 studies were excluded as they focused on the ACC in general, not SACC specifically. Similar case reports were removed, and unfinished clinical trials were also excluded.

The eligible studies for review were 163, including 157 from PubMed and six clinical trials from Cochrane Library.

### 3.1. Data Extraction

All results were evaluated, and information relevant to this study (i.e., epidemiology, clinical features including features of metastasis, histopathology, etiology, diagnostic processes, management; surgery, radiotherapy, chemotherapy, prognosis, novel therapies, and the possible future trends) was extracted.

### 3.2. Epidemiology

Adenoid cystic carcinoma (ACC) is a histopathological subtype of the epithelial malignancies that affects the exocrine glands in the head and neck area and, to a lesser extent, other organs in the body such as the esophagus, uterine cervix, lung, and breast [1, 2]. Salivary gland adenoid cystic carcinoma (SACC) (also known as adenoid cystic carcinoma of salivary glands or ACCSG) is a tumor that originates from both major and minor salivary glands. It primarily affects the minor salivary glands, with the palate being reportedly the most common site, but it can also affect the major salivary glands, where the parotid gland is involved in most cases, followed by the submandibular. Salivary adenoid cystic carcinoma is a rare disease that accounts for approximately 1% of head and neck malignancies and around 10% of salivary glands neoplasms, making it one of the most common salivary gland cancers [2, 3]. There are no reports of a geographical area where the disease is prevalent. A retrospective study, undertaken in the US and analyzed 30 years of data from the National Cancer Institute, showed that SACC has a predilection to the white Caucasian population and a female-to-male ratio of 3 : 2 [2]. However, some small-sample and unicentric studies have reported a male-to-female ratio of 1.6 : 1 [3]. The neoplasm can affect any age group, but it predominantly manifests between the 6th and 7th decades of life which has estimated that there are between 1,450 and 1,660 new SACC cases per year in the USA [2, 4]. However, the incidence of the disease showed a significant decline between 1973 and 2007 [2], which could be attributed to the early recognition resulting from the advances in the diagnostic and treatment provisions. SACC is considered a variant of salivary gland malignancies, and the rarity of the disease made the research into its incidence alone difficult and often included in salivary gland cancer studies. A recent systemic review of 141 multicentric, multicountry clinical studies that included more than 25,800 patients found that adenoid cystic carcinoma was the second most common tumor after pleomorphic adenoma and the most common malignancy in the salivary gland [5].

### 3.3. Clinical Features

The clinical behavior of SACC is similar to other malignancies that affect the salivary glands, with no detectable clinical signs or symptoms for lengthy periods, sometimes years [2]. Salivary adenoid cystic carcinoma is an insidious tumor that grows very slowly and can remain unrecognized until it reaches advanced stages. This is precisely accurate for ACC of the minor salivary glands, which commonly takes longer to be diagnosed [6]. Salivary adenoid cystic carcinoma most commonly affects minor salivary glands, particularly the palate, where it manifests as a lump and is associated with difficulty in chewing or swallowing but can also affect the tongue and the floor of the mouth [3, 7, 8]. If the primary SACC lesion involves minor salivary glands of the upper aero digestive tract, it can present as dysphagia or less frequently as dyspnea, cough, wheezing, hoarseness, or hemoptysis. [9]. The parotid gland is the most commonly affected major salivary gland, followed by the submandibular [2]. The tumor generally causes enlargement of the involved gland in the form of a lump or nodule in the periauricular and/or infra-auricular areas, or significant swelling of the affected side of the face sometimes can reach an extensive size if neglected [10]. Furthermore, SACC of the parotid has been reportedly associated with an odontogenic-like pain referring to the maxillary sinus and sialolithiasis [11, 12]. Salivary adenoid cystic carcinoma of the submandibular gland can present as a slow-growing swelling in the floor of the mouth, often interfering with speech and mastication thus readily detectable or in the form of a lump affecting the submandibular area or presenting in the posterior lower border of the mandible on the affected side [13]. Other clinical features which have been linked to SACC of the submandibular gland include hyposalivation due to sublingual gland obstruction [14] and first bite syndrome, a sequela of parapharyngeal space surgery historically linked to the denervation of the parotid gland, not the submandibular [15]. Other clinical features which are frequently associated with SACC include dull pain, altered sensation of the tongue, palate maxilla or face, and/or facial nerve palsy [16], indicating perineural invasion (PNI) of the local nerves, a sign mainly encountered in the advanced stages of the disease [1, 17]. Furthermore, some reports have interestingly linked SACC to ectopic Cushing syndrome. Despite the slow development of the SACC, it is considered an aggressive tumor that can easily invade the surrounding structures. Perineural invasion (PNI) is a distinctive feature of SACC by which the tumor cells travel along the nerves causing distant metastasis, especially intracranially. In a systemic review of 22 studies, PNI was discovered in more than 40% of 1,332 patients diagnosed with SACC and was reportedly associated with poor disease prognosis regardless of the age group [18]. Other factors which may increase the likelihood of distant metastasis include solid histology, tumor size of >3 cm, and the involvement of the regional lymph nodes [1]. It has been hypothesized that SACC cells may differentiate into Schwann-like cells, which facilitates their travel along nerves without triggering a host response, thus remaining undetectable for a prolonged time [19]. Moreover, SACC can spread via the conventional perivascular route, most commonly to the lungs, followed by bone, liver, skin, and breasts, and rarely intracranially. However, intracranial metastasis is likely to occur in other ways, such as PNI, or via direct invasion of the base of the skull by an adjacent primary lesion [20].

#### 3.3.1. Metastatic Features

Salivary adenoid cystic carcinoma is well known for its locoregional aggressive behavior and distant metastasis. The tumor can spread via the conventional route of perivascular perfusion or distinctively by perineural invasion (PNI), a unique mechanism that differentiates this cancer from other similar malignancies that affect the same sites. Several studies have investigated the association between SACC and PNI, and they have reported that approximately 40% to 60% of SACC cases showed evidence of PNI [21]. Although intracranial metastasis of SACC has been frequently linked to its PNI feature, it is extremely rare for the tumor to result in brain metastasis. However, Nair et al. reported a case of SACC of the palate, which presented as an ipsilateral palsy of the 6_th_ cranial nerve and suggested that the tumor may have spread through the cavernous sinus [22], which highlights the importance of using neuroimaging in cases with nontraumatic abducent nerve palsy. Another means of spread that SACC has frequently shown is the dissemination *via* the lymphovascular route. In a preliminary study investigating cervical lymph node metastasis in SACC cases, about 10% of the sample (*n* = 62) had lymph node metastasis at the time of surgery or thereafter [23]. A primary SACC can also spread *via* the perivascular route to other sites, such as the base of the skull, extradural spaces, brain, and scalp [24, 25]. Although PNI is a well-known clinical and histopathological feature of SACC that significantly impacts SACC prognosis, the specific mechanism underlying its pathological development is still unclear [26]. Many in vivo and *in vitro* studies have been conducted to investigate the biological and pathological mechanisms of SACC metastasis and have linked some biomarkers to the development of the metastatic uniqueness of the disease and how these biomarkers may influence the spread of SACC through the nerves or *via* the lymphovascular route [27]. This finding can result in a new approach that can potentially be applied to tumor diagnosis and treatment. An *in vitro* study conducted on mice has established that overexpression of the transcriptional activator MYB, an oncogenic protein from the human myleoblastosis transcriptional family, plays a role in the metastasis of the SACC, especially to the lungs [28]. The study has demonstrated that MYB is aberrantly overexpressed in SACC tissues, which could promote SACC cell proliferation and metastasis, and has concluded that MYB may potentially be a novel therapeutic target for SACC treatment [8]. Furthermore, the findings of Fu et al. [29] suggest that miR-103a-3p may act as a tumorigenesis factor that promotes the distant spread of SACC to the lungs, and the authors have concluded that this biomarker could contribute to the understanding of SACC pathogenesis and provide a new prospect for potential therapeutic targets. Another study has identified that significantly elevated CCR5 levels, a gene-encoded protein that is known to be involved in some human cancers, in SACC tissues were associated with distant metastasis, thus blocking these markers in the primary lesion could potentially help limit the tumor metastasis [21].

#### 3.3.2. Organs with SACC Metastasis

Salivary adenoid cystic carcinoma is an aggressive tumor that spreads both locally and systemically to several organs. In a retrospective study of 74 patients with distant metastasis, the lungs were the single site of metastasis in 50 cases [20]. Metastasis to cervical lymph nodes reportedly affects 5% to 10% of the SACC cases. A large, retrospective Chinese study (*n* = 798) identified cervical lymph node metastasis in 10% of SACC cases [30]. The liver was reported in several studies as a single site of the systemic spread of the disease. Organs with less common metastasis include the breast and larynx, and those which are rarely involved in SACC metastasis include the pituitary gland, the sternum, the dorsal spine [31], the choroid, the toe bones, and the pericardium [31–38].

Furthermore, it has been reported that SACC of the submandibular gland had a higher tendency to metastasize than the parotid, which could be attributed to the ability of the submandibular tumor to generate more tumor-associated blood vessels and tumor-induced angiogenesis [39].

### 3.4. Etiology

The etiology of SACC has insufficiently been reported in the literature, and there is no sound evidence to precisely describe the factors that trigger the tumor. In addition, the precise pathogenesis of the disease is not fully understood. However, the sequence of the disease development has been studied widely using the same approach used to study other types of cancers, but SACC studies were explicitly exploring the potential factors underlying the disease progress and metastasis. Areas that have been extensively reported include genetics, the role of biological biomarkers, the spreading features, and to a lesser extent, the involvement of viruses and bacterial biofilm in the SACC disease process.

#### 3.4.1. Genetics

The research into the potential genetic involvement of SACC has been focusing on examining the same genetic mutations that have already been proven to play a role in the pathogenesis of other cancers. Most of the reported findings have confirmed that genetic factors are potential initiators of SACC. However, there have not been firm findings on which specific gene type or mutation causes SACC. Dai et al. [40] have investigated the possible link between beta-calcitonin gene-related peptide *β* CGRP of rs2839222 T/T genotype and SACC occurrence, and the study findings have concluded that this gene could be a high-risk factor for SACC because the serum levels of CGRP and *β* CGRP peptides were significantly high in SACC patients. It has also been reported that the gene mutation KDM6A could play a role in the SACC disease process [41]. The study has also reported a new gene mutation KRAS in two cases of SACC of Bartholin's salivary glands in the lip. Xie et al. [42] have detected evidence of upregulation of the NOTCH signaling cascade, a well-known signaling pathway that has been proven to contribute to the development of some human cancers, and its genetic receptor NOTCH1, as well as its downstream gene HES1, in the carcinogenesis, invasion, and metastasis of SACC, potentially by promoting the epithelial-mesenchymal transition (EMT)-related genes [42]. While the aforementioned studies have directly investigated specific genes and pathways to ascertain their role in the etiopathogenesis of SACC, a study by Liu et al. has studied the gene expression profiles of the GSE88804 dataset from Gene Expression Omnibus on 22 cases of SACC and normal salivary gland tissues, to investigate and identify the key genes in the development of SACC [43]. Although the study findings were inconclusive on which type of genes and/or genetic mutations were involved in the occurrence and progression of the disease, they have confirmed the upregulation of 119 and the downregulation of 263 differentially expressed genes (DEGs) in SACC samples compared to the normal salivary gland tissues, which strongly suggests clear genetic links of SACC development. This suggestion can be supported by studies of the etiopathogenesis of ACC in other parts of the body. Pei et al. [44] reported that MYB or MYBL1 genes were detected in all samples of trachea-bronchial ACC and concluded that these genes could serve as a hallmark in the occurrence of the tumor. In 2017, Channir published a case report presenting a genetic involvement of (MYB-NFIB fusion) in two cases of ACC of minor salivary glands from a daughter and her father and suggested that SACC patients may have a family history of the disease, especially in first-degree relatives [45]. Furthermore, samples of SACC and normal salivary glands tissues were compared using immunohistochemistry (IHC) staining to determine the role of the inhibitor of DNA binding 1 (ID1) and the gene expression levels of known ID1 target genes, including S100A9, CDKN2A, and matrix metalloproteinase 1 (MMP1) [46]. The results identified overexpression of ID1 and all target genes in SACC samples compared to the normal tissues. The study concluded that ID1 has a significant contribution to SACC oncogenesis, invasion, and metastasis. The results were further confirmed by blocking ID1 activities in SACC cells using target genes, resulting in decreased cell proliferation, local invasion, and migration. Some genes were negatively affecting the tumorigenicity of SACC by suppressing tumor cell activity, proliferation, and migration, such as the cadherin-4 gene (CDH4), which encoded nonepithelial R-cadherin (R-CAD). In a study of 107 samples of SACC and normal tissues surrounding the tumors, Xie et al. [47] found that R-CAD was overexpressed in all paraneoplastic tissues but only in less than two-thirds of the SACC samples. Moreover, the inhibition of CDH4 *in vitro* increased cell activity, and *in vivo* induced the SACC tumorigenicity. These findings confirmed the tumor suppressing role of CDH4 in the pathogenesis of SACC.

#### 3.4.2. Biomarkers

Several studies have investigated the involvement of specific peptides and proteins in the SACC etiology. In a quantitative spectrometry-based study to analyze the protein expression profile in SACC and peritumoral tissue samples [48], more than 40,000 specific peptides and 4,454 differentially expressed proteins (DEPs) were identified [48]. Of which, HAPLN1 was the most upregulated protein and BPIFB1 was the most downregulated. The study emphasized the importance of investigating the effects of these biomarkers on the SACC occurrence and progression. Kerr et al. [49] compared the expression of Kallikrein-related peptidases (KLKs) in SACC and normal salivary gland tissue samples and reported that all 15 serine proteases of the KLKs were detected in both samples, but KLK1, KLK8, KLK11, and KLK14 were underexpressed in the SACC samples [49]. The study concluded that these biomarkers might play an essential role in the early detection of the disease and its prognosis. An immunohistochemistry study investigated the potential role of the hypoxia-induced proteins in the oncogenesis and metastasis of SACC, and 25 samples from both SACC and normal salivary glands tissues were examined [50]. The study indicated that these proteins were produced intratumorally in a microenvironment that lacked a sufficient oxygen supply and were overexpressed in the SACC samples, inferring that they have a potential contribution to the progression of the disease. Using the same technique, a positive contribution to the tumor invasion and metastasis was evident for the extracellular matrix metalloproteinase inducer (EMMPRIN), which stimulated the matrix metalloproteinase (MMP) expression in highly invasive cancer cells [51]. In a systemic review investigating the potential role of the proteoglycans (PGs) produced by the myoepithelial cells in SACCs, Wang et al. [52] reported a positive contribution of PGs in the proliferation and migration of the tumor cells [53]. When the PG synthesis was inhibited, the metastasis and perineural growth of the cancer cells were significantly reduced, highlighting the PGs' potential role in ACC development and pathogenesis. In a comparative study, Jiang et al. studied the influence of the beta tumor growth factor (TGF-*β*) on EMT and reported that TGF induced EMT through a mediator redox protein thioredoxin 1 TXN [54]. The overexpressed TXN in the SACC cells indicated that it could be a potential marker in detecting and treating SACC.

#### 3.4.3. Disease Spread

Since SACC is an aggressive tumor both locally and systemically, many studies have been conducted to investigate the pathogenesis behind such properties. These studies have shown that various elements contribute to the invasion, metastasizing, and spreading process of SACC, and based on their findings, clinical trials of target chemotherapeutic agents were granted to suppress the disease spread. Many elements were proven to be involved in promoting the invasiveness of SACC. Some of these elements were investigated clinically on samples from patients who underwent surgical resection of SACC lesions. Zhang et al. studied 158 SACC specimens, exploring the potential role of CXCR5 in SACC metastasis [55]. CXCR5 is a protein that has been linked to many human cancers, especially breast cancers with lymph node metastasis. The study identified a significant positive association between the CXCR5 and PNI of the samples. The overexpression of CXCR5 was accompanied by a remarkable increase in the proliferation and migration of the tumor cells. This finding was confirmed by blocking the CXCR5 overexpression, which subsequently suppressed the tumor cells' migration activity and metastasis. The role of the fatty acid synthase (FASN), a known factor in developing cancer, in the development of SACC was investigated in an *in vitro* study [56], which confirmed that FASN promotes the epithelial-mesenchymal transition (EMT), invasion, and metastasis of SACC cells. Blocking FASN resulted in an apparent reduction in the migration and EMT activities of the tumor cells.

Chen et al. reported that a specific molecule, called long noncoding RNA (lncRNA) MRPL23 antisense RNA 1 (MRPL23-AS1), could induce lung metastasis from SACC [57]. Notably, plasma levels of this molecule were markedly elevated in the blood samples taken from patients with SACC, and this was associated with increased EMT and microvascular permeability. Furthermore, many signaling pathways have been identified as potential influencers in developing various human cancers. Ji et al. investigated the role of Claudin-7 CLDN7 in SACC progression [58]. Claudin-7 CLDN7 is a known membrane protein that has been shown to be involved in several signaling pathways that promote some cancers, but its role in SACC is still unclear. This study reported that the protein levels were lower in SACC patients and were associated with an increased level of oncogenic activities of SACC cells. Both EMT and cell migration were attenuated with the overexpression of CLDN7 and were restored when inhibited, highlighting the importance of this molecule in suppressing SACC progression and metastasis. In a similar approach, the role of NR2F1, a protein encoded by the NR2F1 gene which is also involved in many human cancers, was investigated [59]. The study concluded that NR2F1 might be underlying a potential mechanism of the recurrence and metastasis of SACC because its lower expression in SACC samples compared to the normal salivary gland tissues was associated with cancer cell dormancy as well as cell migration and metastasis. An *in vitro* study, that investigated the potential effect of the intertumoral pressure on the oncogenic activities of SACC cells using a simulation of interstitial fluid pressure (IFP), concluded that there was a positive link between an increased level of intertumoral pressure and increased levels of proliferation and migration activities of the tumor cells.

The lack of information reported in the literature regarding the etiology of SACC necessitates further clinical and immunohistochemical studies to understand the exact or potential etiological mechanisms of the disease.

#### 3.4.4. Bacterial and Viral Involvement

The available literature lacks studies that have examined any potential role of bacteria in the etiology of the SACC. However, one study compared the oral bacterial biofilm from 13 SACC patients with samples from a control group of 10 healthy patients and concluded that there is a clear difference in the composition of the microbial flora between the two groups. However, there was no evidence to support any theory underlying the bacterial initiation of SACC [60].

Three studies explored the potential involvement of certain viruses in the development of SACC. Two studies attempted the detection of the viruses in SACC samples and patients, and the third study explored the role of some viruses in the disease development. Viruses investigated were a polyomaviruses group of three viruses (SV40, JCPyV, and BKPyV), human papillomaviruses HPV, and Pim-1 siRNA.  Table 1 summarizes the findings of the three studies.

### 3.5. Diagnosis

#### 3.5.1. Diagnosis of SACC

The definitive diagnosis of SACC usually requires clinical examination, radiographic investigations, and histopathology.

#### 3.5.2. Clinical Examination of SACC

Salivary adenoid cystic carcinoma generally presents as a slow-growing lesion of the affected salivary gland. But this feature is not distinctive, and most of the lesions show unsuspicious clinical appearance. In rare cases, however, the clinical presentation does raise the suspicion of malignancy, but the presentation is not specific for SACC.

Clinical assessment of the site is usually the first step in the diagnosis. When the overlying mucosa shows no abnormal properties, the clinical significance of any underlying clinicopathological feature of the SACC will be obscured; hence, the diagnosis based on the physical examination will be challenging. If the clinical presentation suggests a malignancy due to abnormal soft tissue presentation, bleeding, and necrosis in the mouth or PNI-related symptoms, SACC should always be included in the differential diagnosis until proven otherwise.

### 3.6. Radiographic Assessment

#### 3.6.1. Ultrasound

The first line of imaging when suspecting neoplastic growth in a major salivary gland is ultrasound [61], which is a noninvasive technique that can help identify the nature of any lump, its approximate borders, and its contents [62]. Additionally, ultrasound can be used in some diagnostic or interventional procedures as a 3D guidance when performing fine needle aspiration biopsy (FNA) or cytology screening for SACC as an initial biopsy technique.

#### 3.6.2. Computed Tomography CT and MRI

Computed tomography (CT) (or contrast-enhanced cervical computed tomography-CECCT) is crucial for detecting any bone involvement and accurately determining the tumor borders. The reported CT features of SACC are variable, usually dependent on the site, the stage of the disease, and the grade of the bone involvement. In a study of 102 cases of SACC of the palate, Ju et al. [63] reported that tumors showed bone destruction including palatine, maxillary, and nasal bones, enlargement of the greater palatine foramen (GPF), and involvement of the pterygopalatine fossa, foramen rotundum, and cavernous sinus. Magnetic resonance imaging (MRI), contrast-enhanced cervical computed tomography (CECCT), and/or contrast-enhanced MRI (CEMRI) are all essential techniques for diagnosing SACC. They greatly help determine the tumor features accurately, including their effects on the surrounding structures, bone, and soft tissues. Wang reported using contrast-enhanced CT to examine the resection margin after surgery, Wang et al. [64], while Shimamo reported to use CECCT and CEMRI to study the perineural invasion PNI of ACCs in the oral and maxillofacial region [65]. His findings confirmed the usefulness of using both techniques in detecting SACC spreading features with a slight superior accuracy for the MRI. However, he concluded that both techniques are equally valuable for detecting SACC. The use of PET-MRI and/or PET-CT is also critical when looking for metastasis of SACC in the whole body. For instance, Shah reported a case of SACC that had metastasized to the kidney and was subsequently recognized by the PET-MRI scan [66]. Moreover, there seems no significant difference in the diagnostic accuracy between the two techniques, as reported by Ruhlmann et al. [67], who concluded that PET-CT and PET-MRI had an equal accuracy of 94% in detecting local lesions.

#### 3.6.3. Fine Needle Aspiration (FNA) Biopsy

It is a limited diagnostic tool used as an initial, relatively noninvasive procedure when the clinical and radiographic features do not suggest an advanced disease [68]. The technique aims to examine the nature and origin of the cells inside the lumen of the cystic lesion and can either be performed as a simple biopsy under local anesthesia when the cystic lesion is superficial or easy to access or under regional or general anesthesia with ultrasound guidance, ultrasound-guided FNAC [69]. Tummidi et al. [70] reported a case of sinonasal adenoid cystic carcinoma (SNACC) that was successfully diagnosed using FNA cytology alone. The collected specimen showed cell block and positive immunohistochemistry for CD-117, a feature of SACC.

#### 3.6.4. Histopathology

Salivary adenoid cystic carcinoma is a histopathological subtype of the basaloid malignancies that affect the exocrine glands in the head and neck, mainly the salivary glands. The cancer was previously known as “cylindroma” due to its histologic pattern that consists of cylinders of glandular epithelial cells immersed in a dense hyaline stroma [10]. There are three main histopathological patterns for this tumor: cribriform, tubular, and solid. All these subtypes can be identified based on the dominant shape and arrangement of the epithelial secreting cells, the myoepithelial cells, and the extracellular matrix. There is no proper protocol to distinguish between these subtypes, but a recent study has suggested that the histologic subtype can be considered to be solid when the solid pattern accounts for more than 30% of the tumor [71]. A retrospective study of 87 SACC cases indicated that the cribriform subtype was the most encountered histologic pattern of SACC and that the solid was the least common [72]. The study authors used these histopathological patterns to compare the clinicopathological and prognostic features associated with each subtype and concluded that the solid pattern had the least differentiated cells and the richest extracellular stroma, which was the most locally aggressive, with the highest occurrence of PNI, and that it had the poorest prognosis. Belulescu et al. [3] reported that the solid pattern was encountered in 46% of the cases, which contradicts observations from other studies, in which the solid pattern was the least common. But these discrepancies can be attributed to several factors, including the study sample and/or design and the population type. The electron microscope and immunohistochemistry studies of SACC specimens showed two types of cell differentiation: glandular and myoepithelial, with the latter tending to be more dominant. Cells in all histologic patterns show hyperchromatic nuclei and minimal cytoplasm. The histopathological pattern of the cribriform subtype consists of islands of basaloid cells surrounded by spaces that imitate cystic formations. Multiple cyst-like formations with various sizes create a histologic picture that resembles a unique shape of the “Swiss cheese.” Despite the shape of a cyst, these formations are not true glandular cysts and have no lumina. However, true glandular lumina with cuboidal cells can be seen diffused throughout the tumor as microcystic spaces, usually filled with pink and bluish materials that include basement membrane constituents, such as proteoglycans that are usually produced by the glandular epithelium [73]. The histologic picture is nearly the same for the tubular pattern, with a slight increase in the hyalinized extracellular stroma and a formation of nests of the cancer cells rather than cyst-like lesions. In the solid pattern, the cancer cells form random isles with no tubular or cystic formations and the stroma is predominant [73]. The traditional histopathological examination of SACC samples may not be sufficient to reach a definitive diagnosis in some cases; thus, the use of other techniques such as immunohistochemistry may sometimes be necessary. Immunohistochemistry is a histopathological staining technique that uses specific tissue biomarkers to detect neoplastic activities. Despite the insufficient data reported in the literature regarding this diagnostic tool, it has been reported that the expression of biomarkers such as CD-117, P-53, and Ki-67 in a suspected SACC biopsy can precisely differentiate the cancer from its closest imitators [74]. Goulart-Filho et al. [75] used immunohistochemistry to investigate the role of the pathological formation of new blood vessels as a potential mechanism of SACC progression and metastasis and concluded that SACC development is unrelated to neo-angiogenesis. Both immunohistochemistry and histopathological examinations of SACC specimens show biphasic elements in the development of the tumor, which are myoepithelial cells and glandular/secreting epithelial cells, with the former being predominant in most of the SACC cases. A study based on immunohistochemistry labelling indicated that myoepithelial cell proliferation and differentiation in SACC contributed to the disease's carcinogenesis progress more than its epithelial/secreting counterpart [76]. Furthermore, because submandibular ACC often demonstrates more proliferation of the myoepithelial cells and less differentiation than that of the parotid gland, the study concluded that this could potentially explain the aggressive clinical behavior of the submandibular ACC.

#### 3.6.5. The Differential Diagnosis of SACC

The differential diagnosis of SACC requires clinical examination, histopathological investigation, and sometimes immunohistochemistry staining [77]. Clinically, the spectrum of differential diagnosis of SACC is narrow. However, the manifestation of a swelling in a suspected site necessitates the application of a surgical sieve to rule out any other pathology that may present as a lump. Lesions that SACC needs to be distinguished from vary between common and rare and often include neoplasms such as pleomorphic adenoma, mucoepidermoid carcinoma, adenoid basal cell carcinoma, polymorphous adenocarcinoma, acinic cell carcinomas, and myoepithelial carcinomas. Furthermore, a case report found increased plasma levels of IgG in ACC patients and concluded that there might be a link between IgG4-related disease (IgG4-RD) and ACC; thus, the malignancy should be included in the differential diagnosis when encountering IgG4-RD cases [78].

#### 3.6.6. SACC and Pleomorphic Adenoma (PA)

Clinically, there are no specific features that can distinctively differentiate the two neoplasms. Both tumors can demonstrate a similar clinical picture of a painless, slow-developing mass of different sizes, although PA can reach extensively large sizes, which, on examination, tends to be firm, unilateral, well defined, and relatively mobile [79]. Histologically, the primary difference between PA and SACC is the rich plasmacytoid appearance of individual tumor cells in the former, which is considered a reliable feature to differentiate between the two neoplasms [79]. Histologic examination is crucial to differentiate between the two neoplasms as this will eventually affect their prognosis and management; PA is benign, while SACC is an aggressive malignancy.

#### 3.6.7. SACC and Mucoepidermoid Carcinoma (MC)

Mucoepidermoid carcinoma is a rare malignancy that commonly affects the parotid gland. The clinical presentation of MC is similar to its imitators, including SACC. It can present as a solitary lesion of a cystic nature, and its diagnosis is often challenging even on the cytomorphology level [80]. When the histological picture of MC contains the typical cytomorphology of islands of bi-layered epithelium with oncocytic and basal cells, squamous cells showing atypical nuclei, and necrotic stroma, the diagnosis is often straightforward. However, in most cases, these typical features are unclear, which necessitates the use of other techniques such as immunohistochemistry, CT imaging [77], and molecular profiling to reach a definitive diagnosis [81]. All other neoplasms in the differential diagnosis of SACC require a comprehensive diagnostic approach using clinical assessment, histopathology, imaging, immunohistochemistry, and molecular profiling to reach an accurate diagnosis, which is often challenging and requires a multidisciplinary approach.

#### 3.6.8. Grading of SACC

There is no specific system to grade and classify SACC, but a general system (Milan system) is used to report and stratify all salivary gland neoplasms [82]. For the staging and grading of SACC, the TNM system is still used. A recently published study has suggested a more objective system to evaluate the solid components of SACC, a system called minAmax (minor axis maximum) [83]. They identified minAmax as “the length of the minor axis of the maximum estimated oval fitting the largest solid tumor nest in each ACC case.” It is a simple yet effective prognostic tool that can predict the overall survival (OS), disease-free survival (DFS), and disease metastasis-free survival (DMFS) in SACC cases based on a simple measurement using a microscope equipped with a micrometer to measure the solid component of the tumor's samples. It showed an excellent reproducibility with a cut off of 0.20 mm, lower than any other system used before with a Kappa coefficient of 0.81 higher than any previous system. However, the system has some drawbacks, such as it is not helpful in small-sized lesions, and it is not considering other components of the SACC specimen apart from the solid components.

### 3.7. Management and Prognosis

#### 3.7.1. Treatment of SACC

Until today, there is no standardized comprehensive treatment that can be used in managing all SACC cases. The available literature lacks high-quality reviews of the current management approaches, outcomes, and long-term follow-up. Generally, management of SACC depends on the size of the primary lesion, metastasis and the grade of the disease, and the patient's general health, and it often consists of a combination of surgery, postoperative radiotherapy (PORT), and, occasionally, chemotherapy.

#### 3.7.2. Surgery

The current surgical regime still comprises tumor resection with safe margins, with or without reconstruction. Surgery should be planned according to the size and the site of the lesion, and the surgeon should ensure that the tumor is both accessible and resectable. Surgery is still the first line of treatment when the tumor is resectable, but postoperative radiotherapy is required in some cases, such as large lesions, lesions with postoperative positive or close margins, and nonresectable lesions. However, the effectiveness of PORT for smaller tumors is still controversial. In a retrospective study of 58 SACC cases in which surgery was the primary treatment in more than two-thirds of the cases, the 10-, 20-, and 25-year survival rates were 63.7%, 27.3%, and 20.0%, respectively [84]. Many case reports and retrospective studies have indicated that surgery is the most effective treatment approach compared to other treatment options such as radiotherapy and chemotherapy. However, surgery has some limitations that can reduce its effectiveness, as its application and effectiveness often depend on the location and size of the tumor, the experience of the surgeon, and the provisions at their disposal [85]. Although negative margins are associated with better overall survival rates and disease-free progression [85], complete tumor resection with negative margins is not always possible. Therefore, PORT is sometimes needed to compensate for the incomplete removal of the tumor. High rates of postoperative positive margins have frequently been linked to poor prognosis and increased likelihood of recurrence, especially in patients who do not receive a PORT [86]. It seems that, in many cases, standalone surgery is insufficient to establish a better prognosis and survival rate and that surgery with PORT can achieve better outcomes [87]. In a recently published systemic review of the current treatment approaches for SACC, Ran et al. have reported that surgery was the sole module of treatment in over 40% of the sample, surgery with PORT in 35%, while standalone radiotherapy was used in 19% of the cases [9]. The study has investigated the outcomes of the two most used modules in the treatment of SACC, i.e., surgery alone and surgery with PORT, and has reported that the 5- and 10-year survival was better in the second module: 86.4%, 55.6% and 97.3%, 44.4%, respectively. In a systemic review of SACCs with metastasis to the lungs, the surgical removal of the metastatic lesions proved to reduce disease progression and increase the overall survival rate [88]. Nevertheless, the procedure was widely dependent on the condition of the lungs, the size of metastasis, and the patient's general health. Another systemic review which studied the influence of elective neck dissection (END) on the topical spread of the disease and metastasis-free period when conducted together with the surgery concluded that patients who underwent surgery with END showed a better metastasis-free period but recommended that this procedure should be limited to levels I to III of the lymph nodes [89].

#### 3.7.3. Radiotherapy

Radiotherapy alone is seldom used to treat SACC as it has been shown to be insufficient, and it is only indicated in advanced stages and nonresectable cases of SACC [90]. However, the use of postoperative radiotherapy (PORT) as an adjunct modality with surgery is reportedly effective and has become widely used in the management of SACC [91]. Indeed, it has been reported that patients who did not undergo PORT were 13 times more likely to develop local recurrence than patients who received the treatment [86]. Nevertheless, a large retrospective study that analyzed data from more than 4000 SACC cases has indicated that the use of PORT in ACC of the submandibular gland was only helpful in stage III tumors and had no benefit in the early stages [92]. The study has also reported that ACC of the submandibular gland had the worst prognosis compared to SACC of the other salivary glands. There are four techniques reportedly used in delivering the radiation dose to the tissues affected by SACC: 3D conformal radiation therapy (3D-CRT), image-guided radiotherapy (IGRT), brachytherapy, and intensity-modulated radiotherapy (IMRT). All these types are used in PORT, but there is a lack of evidence on which modalities provide the best results when treating SACC, although they have all been linked to improved treatment outcomes and prognosis. A retrospective analysis of 40 cases of SACC reported that the use of both IGRT and IMRT techniques showed no better outcomes than 3D-CRT [90]. Lang et al. [93] studied the application of IMRT with carbon ions as a booster following surgery and reported reasonable control of the local disease and an improved overall disease-free survival (DFS) rate. In a single institute experience of ACC of the parotid gland, iodine-125 interstitial brachytherapy was used as a PORT technique in 86 patients [94]. The results showed promising outcomes: the 5- and 10-year DFS rates were 74.8% and 66.6%, respectively, highlighting the effectiveness and safety of iodine-125 interstitial brachytherapy as a PORT. Moreover, brachytherapy was used for a locally recurrent case of ACC of the tongue, and the technique successfully eliminated the recurrent lesion. Ha et al. [95] and Lee et al. [96] indicated that conjoined radiotherapy and chemotherapy in patients with unresectable SACC effectively achieved complete remission in 80% of the cases [97]. Similarly, Hsieh et al. investigated the use of concurrent chemoradiotherapy following surgery and reported that this approach was effective in controlling local recurrence but was ineffective in improving the overall survival rate. The reported therapeutic dose of the radiotherapy ranges between 30Gy to 70GY, with doses above 60Gy linked to better outcomes in terms of a more prolonged disease-free survival (DFS) when compared to doses of less than 60Gy: 40 + 18.87 months for the former, and only 13 + 3.4 months for the latter [91]. Recently, the application of heavy ion therapy has shown [103] promising results in nonresectable cases and primary tumors near the skull base [102].

#### 3.7.4. Chemotherapy

Chemotherapy alone has little or no influence on the treatment of SACC. Many clinical trials have tested some chemical agents as potential systemic drugs to treat the unresectable SACC, advanced stages, recurrent lesions, or when the other treatment modules have failed to produce any clinical benefits. The results were not consistent, and the effectiveness of this treatment module needs further research with new approaches and novel agents. Some clinical trials are still ongoing, but the available trials are phase I and II only, and they investigated mainly antiangiogenic agents from different generations. The following table summarizes these studies.

Several studies have investigated the exact mechanism of action of some therapeutic agents. Wang et al. investigated the effect of erlotinib on SACC tumorigenesis and concluded that while the drug inhibited some tumor cells' activities, it encouraged others such as cell aggregation and regeneration by promoting stem cell-like potential [53]. Chemotherapy in SACC management has limited use, and the current evidence does not indicate a substantial clinical benefit from using the most common agents. Despite achieving a stable disease in many cases, the overall results are not satisfying.

Generally, an effective systematic therapy to manage SACC is lacking, especially in late stages and unresectable tumors; hence, the need for more clinical, pathological, and genetic studies to understand the carcinogenesis and pathogenesis of SACC, and potentially provide new treatments that target its aetiological mechanisms.

#### 3.7.5. Prognosis of SACC

Both the topical/regional invasion and PNI properties of the disease have been linked to the high rate of recurrences and resistance to treatment. Recurrence of SACC is common, and the risk of this happening after the initial treatment is believed to be as high as 50% in some cases [104]. Such a high risk of recurrence, together with the lack of adequate, comprehensive treatment, contributes to the poor prognosis. Furthermore, recurrent and metastasized adenoid cystic carcinoma (R/M ACC) can resist the treatment for more extended periods and make disease management challenging. Some studies reported a better prognosis in females and younger patients with 5-, 10-, and 15-year survival rates of 90.34%, 79.88%, and 69.22%, respectively [76]. A recent study published in 2020 reported a lower overall survival rate for 5 and 10 that vary between 68% to 80% and 52% to 65%, respectively, but the study sample was small (*N* = 49] [102]. The study also indicated that the long-term survival rate was between 23% and 40%.

It has been indicated that PNI and other factors, including locoregional invasion, are risk factors for recurrence and resistance to treatment and that the risk significantly increases when these factors coexist [104]. Recurrent and metastasized salivary gland adenoid cystic carcinoma (R/M SACC) resulting from PNI and typical spread is often challenging in terms of management and prognosis, and patients may need to undergo several surgeries and postresection radiotherapy [105]. Moreover, recurrence and metastasis are often associated with poor long-term prognosis and disease-free survival [21].

### 3.8. Novel Therapies and Future Trends

#### 3.8.1. Novel Therapy

Until today there is no novel chemo- or radiotherapy that has been approved or applied in the management of SACC, with the exception of some novel approaches that used conventional agents such as concurrent chemoradiotherapy [95, 106], which have previously been discussed in section 3.7.3 of this thesis. However, a novel approach that combines a traditional antiangiogenic drug with immune checkpoint inhibitors, toripalimab and anlotinib, has been shown to be helpful in human cancers, including SACC [55]. The study results have highlighted the effectiveness of these agents in reducing lung metastasis and improving disease stability. Another study that used three drugs together vorinostat, pindolol, and tofacitinib [107] to treat an advanced case of SACC has reported an improvement in the disease stability but only a partial response to treatment. Therefore, surgery and postoperative radiotherapy will remain the mainstay of treatment in most SACC cases for the foreseeable future because most of the novel chemotherapeutic agents have demonstrated limited effectiveness in the management of SACC [108–115].

#### 3.8.2. Future Trends in Diagnosis and Treatment

Many studies have investigated the viability of using specific biological markers, proteins, and signaling pathways that have been shown to promote or suppress SACC as a potential target therapy or as an early hallmark for the diagnosis of SACC. Tables 2 and 3 summarize these studies [97–102, 109, 116–118].

## 4. Conclusion

Salivary adenoid cystic carcinoma is a rare cancer but is one of the most common salivary gland malignancies. The disease is not prevalent, and its etiopathogenesis is poorly understood, although several genetic patterns and biomarkers have been linked to its initiation and/or progression.

The diagnosis is complex and, in many cases, requires special investigations to reach a definitive diagnosis. Management is often challenging, and the disease frequently shows recurrence.

Metastasis surgery and adjuvant radiotherapy are still the first line of treatment, while the effectiveness of chemotherapy is still limited, although it achieves some disease stability in incurable cases and palliative management.

The future trends in the diagnosis and management of SACC depend on the discoveries of certain elements attributable to the disease oncogenesis. However, the rarity of the disease hampers the striving for further research and clinical trials to explore new approaches and novel therapies. The insidious clinical behavior of SACC, its poor prognosis, and its aggressiveness should invite more interest in laboratory and clinical studies to investigate the etiology and the development of the condition.

## Figures and Tables

**Table 1:** Viral and bacterial involvement in SACC.

Virus	Study (author)	Aim	Etiological significance
Polyomaviruses, SV40, JCPyV, and BKPyV	Hanna Hämetoja et al. 2019	Detection of virus in SACC: only JCPyV is detected	No
Human papilloma virus (HPV)	Hong-Xia Liu et al. 2017	Detection of virus in SACC: not detected	No
Pim-1 siRNA	Xin Zhu et al. 2014	Potential negative effect on SACC development	No etiological contribution but can be a target of a new therapy

**Table 2:** A list of recent studies of potential diagnostic and therapeutic agents.

Study/author	Investigated target	Potential use	Comment
(Nightingale et al.) [97]	Prostate-specific membrane antigen (PSMA)	Therapeutic target	
(Qiao et al.) [98]	MiR-140-5p	Therapeutic target	
(Wang et al.) [52]	Globularifolin	Therapeutic treatment	Target/systemic
(Liu et al.) [99]	The synergistic effect of both JQ1 and PI3K	Novel treatment combination	
(Cai et al.) [100]	Simvastatin	Therapeutic target	
(Huang et al., 2018)	HES1	Therapeutic target	
(Chen et al.) [101]	Regorafenib	Systemic drug	
(Ma et al., 2017)	AGR2	Therapeutic target	
(Yang et al.) [102]	SOX10	Diagnostic marker	

**Table 3:** Update of the novel and potential therapies used in treating ACC in areas different from salivary glands.

Study/author	Investigated target	Potential use	Comments
(Doddapaneni et al.) [116]	Fibroblast growth factor receptor 1 (FGFR1)	Therapeutic target	Used for ACC of lacrimal glands
(Udagawa et al.) [117]	Liposomal formulation of eribulin (E7389-LF)	Therapeutic agent	Used for ACC of salivary glands, thymus gland, and other sites
(Andersson et al.) [118]	Targeting the oncogenic transcriptional regulator MYB by inhibition of IGF1R/AKT signaling	Therapeutic target	Used for ACC of salivary glands, ACC lacrimal glands, and other sites
(Tchekmedyian et al.) [109]	The multitargeted tyrosine kinase inhibitor lenvatinib	Therapeutic agent	Used for ACC of salivary glands, lacrimal glands, breast, bronchi, and the external auditory canal

## Data Availability

The data used to support the findings of this study can be obtained from the corresponding author upon reasonable request.

## Abbreviations

ACC: Adenoid cystic carcinoma
ASE: Adverse side effect
DDSACC: Differential diagnosis of SACC
DEGs: Differentially expressed genes
DFS: Disease-free survival
DMFS: Disease metastasis-free survival
EMT: Epithelial-mesenchymal transition
END: Elective neck dissection
FDA: Federal drug agency
GDP: General dental practitioners
GI: Gastrointestinal
IGRT: Image-guided radiotherapy
IMRT: Intensity-modulated radiotherapy
MaSG: Major salivary glands
MFS: Metastasis-free survival
MiSG: Minor salivary glands
MYB: An oncogenic protein from the myeloblastosis transcriptional family
NHS: National Health ervice
NLM: National Library of Medicine
OMFS: Oral and maxillofacial surgery
OS: Overall survival
PORT: Postoperative radiotherapy
R/M ACC: Recurrent or metastatic adenoid cystic carcinoma
RCTs: Randomized controlled trials
SACC: Salivary adenoid cystic carcinoma
SNACC: Sinonasal adenoid cystic carcinoma
UCL: University College London
UCLH: University College London Hospital
WHO: World Health Organization.
